# No longer alone: Primary care physicians’ experiences of the rehabilitation coordinator

**DOI:** 10.1080/02813432.2025.2486145

**Published:** 2025-04-04

**Authors:** Lottie Sällström Randsalu, Kjerstin Stigmar

**Affiliations:** a Primary Healthcare Center Staffanstorp, Region Skåne, Sweden; b Department of Health Sciences, Lund University, Lund, Sweden

**Keywords:** Rehabilitation coordinator, primary healthcare, sick leave, rehabilitation process, insurance medicine

## Abstract

**Background and Purpose:**

Absence from work due to illness is common in many western countries and has many negative consequences for both the individual and society. Since 2020 Swedish healthcare regions are required to provide resources to coordinate the rehabilitation process, a complex system involving medical as well as work-related parties, where both the physician and the rehabilitation coordinator play a central role. The aim of this study was to describe how primary care physicians experience the role of and the collaboration with a rehabilitation coordinator

**Materials and methods:**

We used a qualitative design doing semi-structured interviews with primary care physicians (n= 9) in the Skåne healthcare region. The interviews were recorded, transcribed and analyzed using qualitative content analysis.

**Results:**

One main category “An external and internal connecting point that has improved the sick-listing and rehabilitation process”, and four subcategories: “Provides relief for the individual physician”; “Offers practical support at the clinic”; “Gives increased sense of security for the patients” and “Sufficiently trained, with potential to take further responsibility”, were determined.

**Conclusions:**

The study shows that primary care physicians experienced benefits from a close collaboration with a rehabilitation coordinator, feeling less lonely. The rehabilitation coordinator is often regarded as having a central role in insurance medicine related tasks, but an even more active role is desired.

## Introduction

Absence from work due to illness is a common problem in Sweden [1,2], and many other Western countries [3]. There are many negative consequences with sick leave, such as exclusion from work and society and impaired work ability that further decreases over time [4]. The downward spiral of successively decreased work ability, prolonged sick leaves and increased exclusion is a problem not only for the individual patient but also for the employers and for society as a whole.

In Sweden an individual whose work ability is limited due to an injury or disease is entitled to sick leave benefits [5]. Physicians diagnose the patient and assess their work ability, although it is the Swedish Social Insurance Agency (SSIA) that makes the decision regarding sick leave and sick leave benefits [6,7]. Writing medical certificates is a common task in a primary healthcare setting, and here the physicians play an essential role [8].

Many physicians find insurance medicine related tasks, especially medical certificates, difficult and a significant work-related burden [9–12]. The difficulties arise due to limited training in insurance medicine [13], a high number of consultations each week involving medical certificates, as well as handling disagreements with patients [14]. Historically, due to a lack of organized coordination between the parties involved in the rehabilitation process, patients have been at risk of falling through the cracks and not getting the support they need [15]. Among physicians, the collaboration with the SSIA is often perceived as dysfunctional and as a stressful and very demanding part of their work [9]. Altogether, this makes insurance medicine related tasks a psychosocial work environment problem for many physicians [8,12].

A rehabilitation process is no doubt a complex system and sometimes the involved parties may have differing views on what the best rehabilitation efforts may be [16], and involving multiple actors with different assignments can lead to problematically diverse views on what kind of efforts are best for the individual [17]. Early intersectoral collaboration and integration of involved actors is important for keeping the patient connected to the workplace, where the employer is responsible för the vocational rehabilitation. These strategies are of great importance for a better functioning rehabilitation process and to avoid long and passive sickness absences [18,19].

Primary healthcare has long lacked a formalized coordination of the return-to-work (RTW) process between employees and employers, even though the employer has an important role in minimizing the negative effects of the sickness absence and facilitating the RTW-process [20]. Public investigations have shown that when cooperation between these different actors fail, the risks of prolonged sick leaves increase and the possibilities of rehabilitation and regained work ability decrease [21,16,24].

Both on an individual level as well as on a socioeconomic level, there is a great need to facilitate the RTW-process [4]. To achieve a more rapid RTW-process, different coordinated efforts have been provided by employers, healthcare providers and involved authorities. The objective of these interventions has been to limit the long-term sick leave and its associated negative consequences for the individual, as well as for society as a whole [20]. Through educational programs, conducted in cooperation between primary healthcare and external partners, as well as focusing on salutogenic factors and potential capabilities instead of the patients’ underlying medical conditions, research has shown a quicker recovery and a positive impact on sick leave as well as on health-related quality of life [22,23].

In February 2020, a new law concerning individuals on sick leave was implemented in Sweden, requiring the healthcare regions to provide the resources to coordinate the rehabilitation and the RTW-process through coordinated efforts [24]. The rehabilitation coordinator performs a number of tasks, where the three major assignments are internal collaboration, supporting the patient, and collaboration with external parties [25]. In general, the rehabilitation coordinator is a professional already employed at a healthcare unit as a nurse, occupational therapist, physiotherapist, or social worker. The assignment as rehabilitation coordinator is most often part-time. In the healthcare region Skåne, basic education (3 days) is required before entering practice and further education/support for the assignment, is provided by the healthcare region on a regular basis. Academic studies at an advanced level are available (7.5 credits).

Although the use of the rehabilitation coordinators in recent years has received an increased amount of attention, the effectiveness of their work is still not fully established. A recent study indicates that the involvement of a rehabilitation coordinator may be beneficial in the process of getting patients on long-term sick leave back to work [26]. On the other hand, a previous review concluded that there was no evidence that coordinated RTW – processes actually facilitate the process, compared to usual care [27]. In support of the rehabilitation coordinator’s role, other studies have shown a positive association of providing a rehabilitation coordinator and that these coordinated individualized efforts, are of great importance in the rehabilitation process [28,29]. Other previous reviews have also shown the importance of including actors from the workplace at an early stage to reach a more successful rehabilitation process [28,30–32].

In light of all of the above, there is a need to gain a better understanding of coordinated rehabilitation efforts. Since physicians are essential in insurance medicine related work in general, and in medical certificate-related tasks in particular, there is a need to better understand their specific perceptions of a rehabilitation coordinator, a function that they collaborate frequently with, as well as depend on a lot, regarding these complex issues. Therefore, the aim of this study was to study how physicians in primary healthcare setting experience the role of, and the collaboration with, a rehabilitation coordinator.

## Materials and methods

### Design

We did an interview study, applying a qualitative content analysis. The study design was developed through close collaboration between the two authors (LSR: MD, GP; KS: RPT, PhD, associate professor and senior lecturer). The interviews reflect the different physicians’ unique experiences of collaborating with a rehabilitation coordinator as well as their view of the rehabilitation coordinators’ work [33,34]. We used COREQ check list as guidance when planning, conducting and reporting the study [35].

### Selection and recruitment process of study participants

The study was conducted in the Skåne healthcare region, Sweden, in a primary healthcare setting. The study applied a strategic sampling procedure to obtain as wide a range as possible regarding the participants’ opinions of the collaboration with a rehabilitation coordinator [36]. Inclusion criteria were that all participants should be active either as a resident physician or general practitioner (GP) in the setting of primary healthcare. The aim was to have representative sample, including an equal number of men and women, of varying ages. The physicians would also preferably be working in different geographical and socioeconomical areas. In 2021 we contacted healthcare managers at primary healthcare centers in the Skåne region, who received detailed written information about the study by the second author (KS) and were asked to inform their employed physicians about the study. As a first step, we only contacted healthcare centers having a rehabilitation coordinator that had undergone the academic course in rehabilitation coordination (7.5 credits) and also a half year supervision and training, all in all 18 clinics. Since that did not render enough informants, we broadened the search to include healthcare centers where the rehabilitation coordinator had shorter training, thereby contacting another 110 healthcare centers around the Skåne region. All in all, information was sent to a total of 128 healthcare managers, who in turn forwarded the information about the study to an unknown number of possible participants. The physicians who were interested in participating were noted as such by their healthcare managers, contact between the participant and the first author (LSR) was established and further information about the study was given. When physicians agreed to participate, written consent was made, and an interview was scheduled. The number of possibly interested parties that the first author got in contact with was eleven, but since two of these never answered the following invitations for further information, even after getting reminded, nine of these actually agreed to an interview.

### Data collection

The study was conducted using a semi-structured interview guide, consisting of a predetermined set of open-ended questions covering the areas of interest, as is described in our aim for the study [33] (Appendix). It also included a set of questions about background characteristics, about the informants’ number of working years as a physician, if the physician was a GP or resident physician, and whether the physician had training in insurance medicine. The same semi-structured interview guide was used for all interviews. The guide started with the open-ended question ‘What do you know about the role of the rehabilitation coordinator at your primary healthcare center?’ The semi-structured interview guide was developed in collaboration between the two authors of the study. The questions were not asked in the exact same order at every interview, as the aim for the interviews was to have a conversation about the topic and be open to the respective participants’ views and thoughts [37]. There were differences in the depth of the discussions according to how the participants’ interests varied, but the interviewer made sure that all the main areas were covered in every interview [34]. Due to the COVID-19 pandemic the interviews were conducted digitally, using the Zoom video conference platform. All the interviews were conducted by the first author (LSR), recorded through the Zoom platform, with a duration of between 30 and 60 min. Directly after the interview, the video files were erased, the audio files transcribed verbatim by the first author. Laughing, joking, coughing or hesitation was noted when transcribing the interviews. Subsequently the files were deidentified and saved.

### Data analysis

Data analysis was performed using a qualitative content analysis, reflecting the manifest as well as the latent content [33]. No digital analysis program was applied. The process of qualitative content analysis started by both authors doing a naïve reading through the transcripts, to get an overall insight into the content and to obtain a sense of the whole. Reflections were made and keywords noted during this reading. The interviews were re-read multiple times by both authors to get a better understanding of the content and to get an overall feeling for the content, and the material was discussed continuously [36]. Text that was obviously irrelevant to the aim, such as opening questions and wrapping up, was left out. The remaining mass of text constituted the unit of analysis [33].

The material was divided into meaning units, consisting of meaningful paragraphs. The meaning units were condensed and then labeled with codes. An example of this process is provided in Table 1. The coding process started with the first interview, and went on with the other interviews chronologically, while cross-checking for already used codes, with the intention to reduce the total number of codes without losing in depth [33,34,38].

**Table 1.** Quotations illustrating categories.

Meaning unit	Condensed meaning unit	Code	Subcategory	Category
‘And it is a heavy burden to carry this by yourself, but you don’t. We are two, there is someone you can discuss with and take turns, she says: I can call the patient now and talk about how the SSIA has decided…’	Heavy burden that is shared by two, someone to discuss with and can call the patient	Shared responsibility	Provides relief and support for the individual physician	An external and internal connecting point that has improved the sick-listing and rehabilitation process
‘I do not remember if they said it was one or two days education, which seems short, but might be enough. No idea’	Do not remember anything regarding rehabilitation coordinators education	No knowledge regarding education	Sufficiently trained, with potential to take further responsibility	An external and internal connecting point that has improved the sick-listing and rehabilitation process

Codes were grouped together in sub-categories and later in categories. The different steps were crosschecked for accuracy by the authors. The subcategories were continuously reviewed, and the analysis process went forward and backwards several times while re-evaluating the divisions and categorizations. The authors had an on-going discussion about how contextual factors and their preunderstanding and subjectivity may influence the research process, analysis and presentation of the results [39].

Throughout the process, the authors continuously discussed and reflected upon the results, to achieve credibility. Finally, quotations from different interviews were chosen to illustrate the different categories [33,38]. The results are presented so that no individual informant or healthcare unit can be identified.

## Results

Six of the participants were women, three were men. Two of the participants were resident physicians, the other seven were GPs. Most of the participants were in their late fifties, although the youngest were in their mid-thirties and the oldest were soon to retire. Five of the nine informants worked in small villages or in the outskirts of smaller cities, and the remaining four worked in one of the two bigger cities in the Skåne region.

One main category, with a total of four subcategories, was determined during the analysis of the data collected (Table 2). The main category was that the rehabilitation coordinator was described as ‘*An external and internal connecting point that has improved the sick-listing and rehabilitation process*’, being of great value for the individual physician when dealing with complex insurance medicine-related tasks. The subcategories were as follows: ‘*Provides relief for the individual physician”, “Offers practical support at the clinic’*, ‘*Gives increased sense of security for the patients’ and ‘Sufficiently trained, with potential to take further responsibility’.*

**Table 2.** Main category and subcategories.

An external and internal connecting point that has improved the sick listing and rehabilitation process			
Provides relief for the individual physician	Offers practical support at the clinic	Gives increased sense of security for the patients	Sufficiently trained, with potential to take further responsibility

### An external and internal connecting point that has improved the sick-listing and rehabilitation process

The rehabilitation coordinator was described as a central professional and a great resource in insurance medicine and rehabilitation, both in favor of the physician, the patient as well as for the healthcare unit. Many metaphors were used to describe how internal and external collaboration was coordinated and improved.

*I perceive them [the rehabilitation coordinator] sort of like the spider in the center of the web, that they [the rehabilitation coordinator] can keep in touch with all the involved parties and also inform the patient of the whole sick leave process.*

The rehabilitation coordinator was also seen as ‘the missing piece of the puzzle’ and had improved the sick-listing process, so it went more smoothly and efficiently. The physicians experienced that the organization of the work with these questions had significantly improved and there was not an option to go back to before having access to a rehabilitation coordinator.

*Things have worked out more smoothly and effectively, the appointments are reserved as they should. Things that not necessarily should reach the physicians, well, don’t.*

Physicians expressed that they were able to work more efficiently, targeting tasks that require a physician’s competence, since all insurance related work that was not strictly the physician’s responsibility was coordinated by the rehabilitation coordinator.

### Provides relief for the individual physician

Sick listing was described as complex and difficult. Sometimes also emotionally exhausting and overwhelming for the physicians. The physicians felt a lot of pressure. The rehabilitation coordinator was experienced as lighten the load from the individual physician. This relief was not only about the amount of work but also emotional support. Individual physicians were struggling with sick leave and rehabilitation back to work, and now they had a partner to discuss with. They felt less lonely, and the overall stress of insurance medicine related tasks was reduced. This impacted how the work environment was experienced.

*I think that it is an enormous relief to work with the rehabilitation coordinator.*

*I feel that I use my time in a more directed way now, I mean, where it is needed. […] Then there is also this feeling of no longer being alone in these matters.*

Working in a team that included the rehabilitation coordinator was also described as having drastically changed how the insurance medicine-related work was perceived, especially that the collaboration with the rehabilitation coordinator made the physicians feel less lonely.

### Offers practical support at the clinic

The practical insurance medicine related tasks for physicians were described as clearly eased by the collaboration with the rehabilitation coordinator, often in the shape of regular rehabilitation conferences. The rehabilitation coordinator was often the one who set up and took the lead in these meetings. Different occupational categories participated in these meetings, the set-up differing between the different healthcare centers, but most often included physiotherapists, psychologists, counsellors, physicians and the rehabilitation coordinator. Working as a team was perceived as greatly facilitating the physicians’ insurance related work.

*Then all of us get a chance to describe our viewpoint and then we discuss and we make a plan that we document in the medical records and most often one feels very content about it.*

The collaboration, apart from facilitating for the physician when getting stuck or just needing to discuss a problem, was also described as having an educational benefit for the younger as well as more senior physicians. Often the rehabilitation coordinator was able to support with concrete advice.

*Then there is sometimes, that you get a bit of tunnel vision and get stuck, and then it’s nice to just look at it and see, am I thinking about this the right way, can I do something else, no maybe I can’t or maybe I can. And then you elaborate on that.[…] Or if one is insecure, just to discuss it.*

The rehabilitation coordinators added actual and practical values by providing checklists, questionnaires and information in advance. Furthermore, by making sure that different types of medical certificates were handed over to the right authorities and keeping track of how the sick leave period developed. Support with communication with authorities and employers was very much appreciated by the physicians.

Almost all interviewed physicians stated that the actual workload had markedly decreased since the collaboration with the rehabilitation coordinator began, through practical support in insurance medicine related tasks. The insurance medicine related duties were described as the hardest part of the physicians’ work and positive experiences of the practical support from the rehabilitation coordinator were expressed throughout the interviews, especially when dealing with the most complex and difficult patients. The collaboration with the rehabilitation coordinator was described as a source of much needed support when dealing with especially complicated matters

*it is, well, really…there is also a big difference, because many of these matters are hard and difficult, people who never will get better or (sighing)… anyway…there are things in this patient’s life that aggravates the rehabilitation and like…*

*…it is obvious that we often actually get a little bit further, even if one doesn’t think that way beforehand.*

Together with the rehabilitation coordinator, teamwork was more common. Even when dealing with complex matters, things were described as moving forward more easily in the rehabilitation process.

### Gives increased sense of security for the patients

The rehabilitation coordinator was described as an important professional, not only for the physicians, but also for the patients. By facilitating the sick leave and rehabilitation processes, they provided an increased sense of security for the patient. Often the patients did not know how the system worked, and the rehabilitation coordinator had time to explain the sick leave and rehabilitation process in depth, which the physicians often didn’t have that possibility in an overloaded schedule.

*…that these patients, that sometimes nobody wants to care that much about…because too annoying, like… […] …she is sort of also the patients’… lawyer maybe is a bit much…but sort of…she is our patients’ spokesperson sometimes, and that is like really good.*

The rehabilitation coordinator’s role and assignment include being support for the patient, which made the patients feel safer and less vulnerable when on sick leave. The physicians in this study experienced this as much appreciated by the patients, since being on sick leave includes a lot of contacts and obligations for the patients.

*So, there is…she is involved very early – not when everything already has gone bad…she is…she has a very supportive function. […] That’s, she is of great support for us but it seems like for the patients, they practically think she is their big sister.*

To have the rehabilitation coordinator involved early in the rehabilitation process was described as beneficial for the patient. The rehabilitation coordinator could support the patients throughout the whole rehabilitation process and shorten the time for return to work. In insurance medicine the cooperation between physician and rehabilitation coordinator is important since they have different backgrounds, tasks and responsibilities. For example, regarding the issue of sick leave, the physician always has legal liability and thereby the final say, if there would be divided opinions between the two.

### Sufficiently trained, with potential to take further responsibility

The rehabilitation coordinators’ education or training was not familiar to the physicians. They had no knowledge regarding the type of training, content or how long the actual training was. Despite this, the rehabilitation coordinator was experienced as a competent and supportive professional, with sufficient training and background. Furthermore, the rehabilitation coordinators were described as a priority for the healthcare region.

*I do not remember it they said it was one- or two-days education, which seems short, but might be enough [laughs]. No idea.*

In general, the rehabilitation coordinator had the assignment part-time and combined it with their basic profession. They were often already employed as nurses, occupational therapists, physiotherapists or social workers and thereby a well-known colleague at the primary healthcare center. Many also voiced that there would be much to gain by expanding the collaboration between physicians and the rehabilitation coordinator, especially if the regulations regarding sick leave would get stricter over time.

*Well, but then more time [for the rehabilitation coordinator] to be able to have a more active role…in…uhm…the sick leave process, in the rehabilitation processes. Because this is not all about just medical certificates, it’s about rehabilitation in the best possible way.*

There were expressions regarding how the insurance related work could become even more effective if the full capacity of the rehabilitation coordinator could be put to even better use, and that a more active role for the rehabilitation coordinator would not only be advantageous for the physicians, but also for the patients.

## Discussion

In this study we found that the rehabilitation coordinator was perceived as a great source of support for both the physicians and their patients, and also that the rehabilitation coordinator was perceived as having a central role at the primary healthcare center, but that their role could be further developed. Also from a healthcare organizational perspective, the rehabilitation coordinator has potential to make the sick listing process more efficient. In a recent study, managers in primary healthcare described the rehabilitation coordinator as an important part in insurance medicine issues [40].

The purpose of this study was to investigate how physicians in a primary healthcare setting experience the role of, and the collaboration with, a rehabilitation coordinator. As mentioned earlier, there were different reasons for exploring this. On the one hand, it was because previous research has shown contradictory findings regarding the provision of a rehabilitation coordinator [19,26,27]. On the other hand, it was because the Swedish law concerning coordinated rehabilitation processes, where the rehabilitation coordinator has a central role, is still relatively new, as well as the insurance medicine-related work is perceived by many physicians as one of the most burdensome parts of their work [9,12].

In our study, the rehabilitation coordinator was recurringly regarded as a great source of support – to both patients and physicians. Being viewed as a kind of patients’ advocate was perceived as something very much needed, since being on sick leave is often a vulnerable situation in itself. These results are in line with a previous interview study with patients on sick leave due to mental health problems [41], where they also found that the rehabilitation coordinator could be a resource in contact with employers. At the same time, the rehabilitation coordinator was described as having a very positive effect on physicians’ workload, both practically and emotionally, by decreasing the insurance medicine related workload. Many of the interviewed physicians wanted to give the rehabilitation coordinator an even more prominent role, since the collaboration between the rehabilitation coordinator and the physicians was viewed as advantageous and facilitating the whole sick leave certification process. In a recent interview study with healthcare professionals in psychiatric care [42] they concluded that the rehabilitation coordinator was an important part of the patients’ care and not only rehabilitation back to work.

Previous research has shown a need for developing knowledge and skills for physicians regarding insurance medicine related tasks in general, and sick leave-certificates in particular [13]. This viewpoint is repeatedly confirmed in our study. Previous studies have also shown how physicians more often than not experience the sickness certification process as both difficult and problematic [12]. The physicians in our study repeatedly expressed that dealing with insurance medicine-related tasks was a troublesome burden, and that the sickness certificate process was both problematic and draining. These findings are in line with earlier studies, where physicians experience negative and conflicting feelings to insurance medicine-related tasks in general, and to sickness certificates in particular, due to the complexity of assessing a patient’s work ability and possible need of a sickness certificate [8,12,43,44]. Our study emphatically confirms this account of insurance medicine as being a complicated and burdensome part of primary healthcare physicians’ work. It also acknowledges the relieving effect the collaboration with the rehabilitation coordinator has for the individual physicians.

Already in 2008, long before the new law requiring cooperation with a rehabilitation coordinator in the rehabilitation process was implemented in Sweden, a study described physicians’ feelings of lacking support and advice in insurance medicine-related issues [12]. Our study confirms that physicians still feel the need for support in this complex line of work, and even though the new law has been implemented and collaboration between physicians and rehabilitation coordinators has been made mandatory, a majority of the physicians in our study call for a more extended cooperation with the rehabilitation coordinator. None of the interviewed physicians actually knew what kind of education the rehabilitation coordinator had received, but all of them viewed their rehabilitation coordinator as competent and accomplished. At the same time, there were expressions of needing the rehabilitation coordinator function to be further developed into a more active role, to facilitate an even better rehabilitation process. The need for physicians to know exactly what kind of education their rehabilitation coordinator has received might not be as important as their knowing when and how to collaborate with the rehabilitation coordinator in the most efficient way in insurance medicine related work.

In this study, the participants expressed that they experienced that the rehabilitation coordinator had positive impact on the RTW-process, but in a recent study [19], no significant improvement was found in the RTW-process while being coordinated by a rehabilitation coordinator, although the studies differ not only in size and data collection (multiple questionnaires vs our study’s few interviews), but also in the actual setting, where hospitals and institutions were included as well as primary healthcare centers in the Norwegian study [19], thereby making it difficult to compare the findings. A previous study described how the primary healthcare centers lacked a tradition of coordinating the rehabilitation process for patients on sick leave and their, often difficult, process to get back to work [20]. That study was carried out before the 2020 law, and since the implementation of the law different forms of collaborations have emerged, some working better than others. Our study confirms the need to gain a better understanding of how to best utilize the full capacity of the rehabilitation coordinator, not only for getting the patients back to work as efficiently as possible, but also for easing the physicians’ work as much as possible.

To the best of our knowledge, physicians’ perceptions of the rehabilitation coordinator’s role and function after the implementation of the new law in 2020 has not previously been explored in depth. Therefore, this study has aimed to shed light on the individual physicians’ perception of the role of, and the collaboration with, the rehabilitation coordinator since the collaboration has been made mandatory. This study has shown many gains, for both physicians and patients, by a close collaboration between the rehabilitation coordinators and the physicians. Consistent with most of the previous research as well as with the results of this study, we recommend that all primary healthcare centers are given both the time and the space for working out how to organize a collaboration that works best for their healthcare center and their patients’ specific needs.

### Strengths and limitations of this study

This study was strengthened by using the COREQ checklist [35] as guidance when planning, conducting and reporting the study. The research process is described in detail, to enable the reader to judge credibility. We used a strategic sampling procedure to provide a selection of participants with a wide area of experience in primary healthcare. The invitation was wide, reaching more than a hundred different healthcare centers and by that, hundreds of individual physicians. The final informants were both men and women of varying ages, with varying experiences due to working in different geographical and socioeconomic areas, as well as in cities of different sizes. We believe that we have obtained a representative group of informants. A shortcoming might be the number of participants (nine) and furthermore the risk for selection bias, when those who agreed to participate might have had more positive experiences from rehabilitation coordinators. Two of the nine physicians were working at the same primary healthcare center, a limitation counteracted by the fact that they had very different personal backgrounds which offered a different range of experiences even though they worked at the same healthcare center.

Data was analyzed by two authors, the first author (LSR) being a GP and the second author (KS) being an associate professor with extended experience in qualitative analysis, which further strengthened the study’s credibility. Both authors were well familiar with the topic of the study. The first author (LSR) deals with sick leave and collaborating with rehabilitation coordinators every week and the second author (KS) has long experience from rehabilitation back to work in primary healthcare and occupational health, furthermore, engaged in education for rehabilitation coordinators in the healthcare region and at the University. Therefore, this subjective factor could be viewed as a strength in terms of a preunderstanding of the topic, making it easier to gather the material, knowing which questions to ask and interpreting the collected material. On the other hand, this preunderstanding might be a possible limitation. The authors have continuously applied self-reflexivity, considering themselves as an active part throughout the research process. We have had an ongoing discussion regarding experiences, preunderstanding and subjectivity, to stay aware of how this impacted the complete research process.

No relevant data has been excluded, nor has any irrelevant data been included. The process has been transparent, the different steps explained in detail: the selection of meaning units and how these have been condensed, labeled with codes, as well as how categories and subcategories have been determined. How findings are grounded in data is secured by providing quotes to each subcategory. All through the process, there has been an ongoing dialogue between the two authors to seek agreement on these steps. Through analyzing the material and data in a structured and transparent way, we believe that we have reached a reasonable degree of credibility and confirmability.

The transferability of this study to other contexts in Swedish primary healthcare could be assumed to be reasonably high, as the situation for patients in a rehabilitation process and/or on sick leave, is similar all over the country. The transferability could also be assumed to be high since we interviewed physicians with different backgrounds from primary healthcare centers in varying socioeconomic environments. Rehabilitation processes and issuing sick leave certificates are common tasks for GP: s in most western countries, why parts of our study might be transferable to other countries as well. In the end, though, it is the reader’s decision whether our findings are transferable to other contexts or not.

## Conclusion

This study shows that primary healthcare physicians described the rehabilitation coordinator as having a central and important role in primary healthcare, when dealing with sick leave. They experienced benefits from a close collaboration with a rehabilitation coordinator, feeling less lonely. Also, the patients benefitted greatly from the collaboration. Even though the rehabilitation coordinator was regarded as having a central role in insurance medicine related tasks, an even more active role is desired.

## Ethical perspectives

All participants have been informed that their participation is voluntary and that they have been able to withdraw at any time without giving reason for the withdrawal. They have also been informed that the study has been done in a way that makes it impossible to identify them or their healthcare units. Written informed consent has been a prerequisite for participation in the study. The data has been deposited at the Department of Health Sciences at Lund University, Lund, and only the second author (KS) has had access to the data. The study was approved by the The Regional Ethical Review Board in Sweden (Reg.no 2019-00054 (2018/1098).

## What this paper adds

The new Swedish law in 2020 concerning rehabilitation coordinators was implemented without a common system or detailed design concerning the collaboration between rehabilitation coordinators and physicians. It has therefore been important to investigate how the specific role of the rehabilitation coordinator has been experienced by those working closely with them, rendering a unique insight into how the rehabilitation coordinator’s role is experienced by different physicians in different primary healthcare centers throughout the Skåne region. This knowledge is of great value in the continuing development of the cooperation between physicians and rehabilitation coordinators, strengthening both the cooperation per se and the role of the rehabilitation coordinator.

## Appendix

### Interview guide (in Swedish)

Inledande praktiska frågor:

- Arbetstitel? Hur länge arbetat som läkare? Försäkringsmedicinskt sakkunnig?

Intervju:

- Vad känner du till om en rehabiliteringskoordinators roll och uppdrag?
- Vad vet du om rehabiliteringskoordinatorns roll på just din vårdcentral?
- Vilka roller har ni på din vårdcentral kring sjukskrivningsärenden? Sjukskrivningsansvarig läkare?
- Hur fungerar samarbetet kring försäkringsmedicinska ärenden på vårdcentral?
- Hur ser samarbetet ut med rehabiliteringskoordinatorn? Återkommande möten?
- Hur ser arbetet med extern samverkan ut?
- Har samarbetet med rehabiliteringskoordinatorn gett positiva effekter? Exempel?
- Vad ser du för svårigheter och möjligheter i samarbetet med din rehabiliteringskoordinator?
- Upplever du sjukskrivningar som en börda i ditt dagliga arbete? Har det förändrats i och med samarbetet med rehabiliteringskoordinatorn?
- Hur ser du på handläggning av sjukskrivningsärenden framöver? Förändrade upplägg, tex ökat samarbete med rehabiliteringskoordinatorn?
- Vad vet du om rehabiliteringskoordinatorers utbildning?
- Vad vet du om Region Skånes utbildningssatsning för rehabiliteringskoordinatorer?
- Vet du vad ”din” rehabiliteringskoordinator har för utbildning bakom sig?
- Ytterligare tankar, synpunkter?

### Interview guide (in English)

Introductory practical questions:

- Professional title? Number of years practicing? Special competence in insurance medicine?

Interview:

- What do you know about the role and assignment of a rehabilitation coordinator?
- What do you know about the rehabilitation coordinator’s specific role at your clinic?
- What roles do you have at your clinic regarding insurance related tasks? A specific doctor responsible for sick leave related questions?
- How do you coordinate insurance related tasks at your clinic?
- How is the cooperation with the rehabilitation coordinator structured? Recurring meetings?
- How do you structure cooperation with external actors?
- Has your cooperation with the rehabilitation coordinator led to positive outcomes? Examples?
- What difficulties and possibilities do you see in cooperating with the rehabilitation coordinator?
- Do you feel that insurance related tasks are a burden in your day-to-day work? Has the cooperation with the rehabilitation coordinator changed that?
- How do you view insurance related tasks going forward? Changed structure, for example increased cooperation with the rehabilitation coordinator?
- What do you know about rehabilitation coordinators’ education?
- What do you know about the Skåne Region’s educational program for rehabilitation coordinators?
- Do you know what level of education “your” rehabilitation coordinator has?
- Further thoughts, reflections?
